# Fully Automated Breast Density Segmentation and Classification Using Deep Learning

**DOI:** 10.3390/diagnostics10110988

**Published:** 2020-11-23

**Authors:** Nasibeh Saffari, Hatem A. Rashwan, Mohamed Abdel-Nasser, Vivek Kumar Singh, Meritxell Arenas, Eleni Mangina, Blas Herrera, Domenec Puig

**Affiliations:** 1 Intelligent Robotics and Computer Vision Group, Department of Computer Engineering and Mathematics, Universitat Rovira i Virgili, 43007 Tarragona, Spain; hatem.abdellatif@urv.cat (H.A.R.); egnaser@gmail.com (M.A.-N.); vivekkr.singh90@gmail.com (V.K.S.); blas.herrera@urv.cat (B.H.); domenec.puig@urv.cat (D.P.); 2 Department of Electrical Engineering, Aswan University, Aswan 81542, Egypt; 3 Hospital Universitari Sant Joan de Reus, 43204 Reus, Spain; meritxell.arenas@gmail.com; 4 School of Computer Science, University College Dublin, B2.05, Belfield, Dublin 4, Ireland; eleni.mangina@ucd.ie

**Keywords:** breast cancer, breast density, deep learning, mammograms, generative adversarial networks, convolutional neural network

## Abstract

Breast density estimation with visual evaluation is still challenging due to low contrast and significant fluctuations in the mammograms’ fatty tissue background. The primary key to breast density classification is to detect the dense tissues in the mammographic images correctly. Many methods have been proposed for breast density estimation; nevertheless, most of them are not fully automated. Besides, they have been badly affected by low signal-to-noise ratio and variability of density in appearance and texture. This study intends to develop a fully automated and digitalized breast tissue segmentation and classification using advanced deep learning techniques. The conditional Generative Adversarial Networks (cGAN) network is applied to segment the dense tissues in mammograms. To have a complete system for breast density classification, we propose a Convolutional Neural Network (CNN) to classify mammograms based on the standardization of Breast Imaging-Reporting and Data System (BI-RADS). The classification network is fed by the segmented masks of dense tissues generated by the cGAN network. For screening mammography, 410 images of 115 patients from the INbreast dataset were used. The proposed framework can segment the dense regions with an accuracy, Dice coefficient, Jaccard index of 98%, 88%, and 78%, respectively. Furthermore, we obtained precision, sensitivity, and specificity of 97.85%, 97.85%, and 99.28%, respectively, for breast density classification. This study’s findings are promising and show that the proposed deep learning-based techniques can produce a clinically useful computer-aided tool for breast density analysis by digital mammography.

## 1. Introduction

Breast cancer is one of the most common causes of cancer mortality in women across the world, caused by abnormal cells that have grown uncontrollably. Those cells may also grow in some places in the human body where they are generally not found. When that happens, the cancer is termed metastatic. Mammography is a standard and most famous radiology tool to detect breast cancer early, possibly before it has outspread. However, investigating these mammographic images is not feasible for every case and highly relies on the radiologist’s experience, leading to many false positives. Craniocaudal (CC) and Mediolateral Oblique (MLO) are the most frequent screening mammographic views that provide the best view of the breasts’ sidelong, which statistically is the most commonplace for pathological changes. The MLO view is one of the standard views captured from the side of a diagonally compressed breast. In turn, the CC mammographic view is obtained from the superior perspective of a horizontally compressed breast. Various risk factors are associated with breast cancer, of which high breast density is a strong and independent risk factor that can cause developing breast cancer in fibroglandular tissues [1,2].

The study of Astley et al. [3] shows that the subjective assessment of breast density is a more reliable predictor of breast cancer than other automated and semi-automated methods. Besides, it is debated whether mammographic density gives rise to more aggressive cancers. Thus, the influence of mammographic density on prognosis should be studied. Mammographic breast density reflects the amount of fibroglandular breast tissue area that appears bright on mammograms, commonly referred to as breast percent density (PD%) [4]. In other words, PD% refers to the amount of white or bright area, as seen on a mammogram.

Dense breast tissue is common, non-fatty tissue and is not abnormal, but dense breast tissue can make it harder to identify breast cancer and be associated with an increased risk of breast cancer.

**Figure 1:**
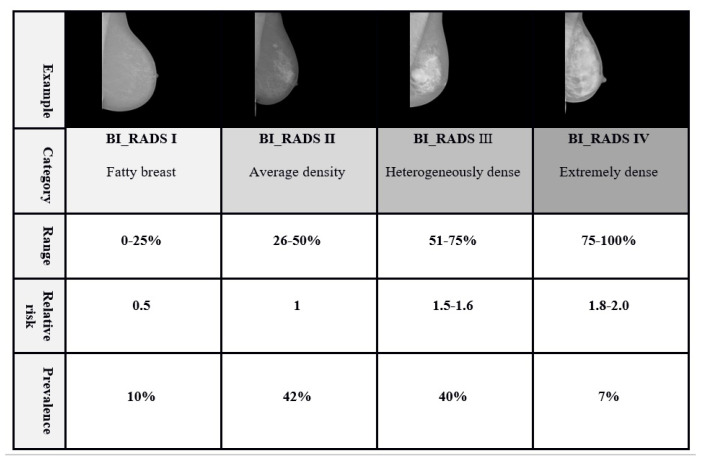
Prevalence, relative risks of developing breast cancer based on Four classes of Breast Imaging and Reporting Data ystem (BI-RADS) density standard (i.e., fatty, scattered fibroglandular density, heterogeneously dense, and extremely dense).

It has been reported that women with a high breast density compared to women with a low breast density have a fourfold increased risk of developing the disease [5]. Figure 1 provides relative risks for developing breast cancer by density category [5].

Diverse computational methods have been proposed in the literature for breast density estimation and classification [6,7,8,9,10,11]. To estimate breast density, researchers have proposed methods to segment the dense region of breasts and divide it by the total area of the breast after excluding the pectoral muscle from the MLO mammograms [12,13].

Numerous image segmentation methods have been used for breast density estimation and classification based on handcrafted feature extraction, such as thresholding [14,15], region growing [16,17], clustering [4], and texture statistical variation [18,19]. However, breast density segmentation and classification are still challenging due to low signal-to-noise ratio and variability of density in appearance and texture [20,21].

Deep Learning (DL), such as Convolutional Neural Networks (CNN), has made several breakthroughs over the past decade, especially in identifying patterns and classifying images. Besides, DL provides several features that other categories of machine learning algorithms do not. Examples of these methods utilized for breast density estimation can be found in the literature [22,23].

The main contributions of this paper are as follows.

- Developing an effective conditional Generative Adversarial Network for segmenting the regions of dense tissues in a mammogram.
- Utilizing the ratio of the dense segmented regions (i.e., resulting in from the cGAN network) to the breasts total area. The computed percentage is used for classifying the mammogram into four different classes of the BI-RADS standard (i.e., fatty, scattered fibroglandular density, heterogeneously dense, and extremely dense).
- Developing a multi-class CNN architecture for breast density classification using the binary masks obtained from the cGAN.

The rest of this paper is organized as follows. Section 2 discusses the related work. Section 3 describes the methodology, and Section 4 presents the results. Last, Section 5 concludes the paper and provides some lines of future work.

## 2. Background Study

In the literature, various breast density segmentation and classification methods have been proposed. The techniques that have been employed to perform these approaches include traditional computer vision techniques and deep learning, which will be reviewed in the following sections.

### 2.1. Traditional Computer Vision Methods

Traditional CAD systems use hand-crafted features based on previous experience and radiologists’ guidance. Handcrafted feature extraction and breast density classification were initially proposed by Wolfe [6]. Wolfe [6] originally described “parenchymal patterns” using verbal descriptions and subjective measures of textural features, and classifies breast density into N1—normal fatty breast; P1 and P2—prominent ducts occupying <25% and 25–75% of the breast, respectively; and Dy—dysplastic breast with sheets of dense parenchyma.

In particular, Cumulus software [24] kept growing the ideas of Wolfe [6] by extending the technology and resources to pursue this line of research. Additionally, Cumulus software [24] is an intelligent tool for understanding breast cancer risk, which is a set threshold for segmenting dense tissue, where regions of breast area are classified into six-category percentages: 0, <10%, 10–25%, 26–50%, 51–75%, and >75%. However, reliance on thresholding might be less accurate, and the significant drawback of threshold-based approaches is that they often lack the sensitivity and specificity needed for accurate segmentation.

In this context, five-grade Tábar classification is proposed by Gram et al. [7], which classifies breast density into five different categories: I—scalloped contours and Cooper’s ligaments, II—evenly scattered terminal ductal lobular units, III—oval-shaped lucent areas, IV—extensive nodular and linear densities, and V—homogeneous structureless fibrosis with convex contours.

Concerning breast density classification methods, well-known breast imaging and reporting data system (BI-RADS) standards have been used [8,25] to reduce complexity in breast imaging evaluation and to aid outcome monitoring.

BI-RADS classification categorizes the breast density into four classes: fatty, scattered fibroglandular, heterogeneously dense, and extremely dense. As shown in Figure 1, they could be ranged from almost entirely fatty tissue to extremely dense tissue with very little fat.

Automated techniques include the LIBRA (Laboratory for Individualized Breast Radiodensity Assessment) software [4] based on multi-cluster fuzzy c-means segmentation produced at the University of Pennsylvania, which is publicly accessible. In LIBRA, a total of 86 features are considered, such as global features like Patient’s age, breast’s thickness X-ray, cluster-merging features like Z-score means, number of unconnected areas, and inter-cluster difference features like compactness and equivalent circular diameter. LIBRA software [4] also produces area-based analyses of the breast area, dense tissue area, and percentage density from full-field digital mammography (FFDM) images. It is a traditional “handcrafted” method for breast density estimation, which determined a 0.81 accuracy, but it has many time-consuming and complicated features.

However, the PD% estimated by the algorithm developed in this study correlates well with BI-RADS density ratings by radiologists and outperforms LIBRA’s algorithm’s accuracy. The accuracy achieved by the algorithm developed in this study is 0.98 for CC-MLO-averaged, significantly higher than LIBRA’s accuracy. The volume-based techniques such as Quantra [26] and Volpara [10] are fully automated software systems to estimate volumetric breast density. Quantra evaluates the thickness of the fibroglandular breast tissue and X-ray attenuation above each pixel in the mammogram images to sum these pixel-wise computations to evaluate the total volume of fibroglandular tissue in the breast. It also evaluates the amount of dense and non-dense tissues at each pixel.

Fully automated methods are currently being developed for obtaining a more objective and quantitative evaluation of breast density. For instance, the Volpara software [27,28] returns the percentage of dense tissue through a volumetric estimation of the breast.

Function only on the raw (“FOR PROCESSING”) digital mammogram images, which are not routinely stored in most medical centers. On the other hand, the semi-automated Cumulus software can display an interactive intensity threshold [24,29], and is rated one of the best methods for achieving a quantitative segmentation [30]. The Quantra method had a slightly higher, but significantly different, correlation coefficient than the Cumulus method for the volumetric breast density correlation between the right and left breasts (*r* = 0.95, *p* < 0.001).

### 2.2. Deep Learning-Based Methods

Recent approaches in machine learning have opened up an opportunity to tackle breast density investigating using deep learning methods. Nowadays, *deep learning techniques* have been used in many studies to automatically extract features from mammograms at multiple levels of abstraction and evidence superior performance. Deep learning networks, such as CNN, can automatically learn features from raw images directly, and they can accurately represent objects at different scales and orientations. The CNN is one of the most popular class of deep neural networks.

For example, Kallenberg et al. [31] investigated an unsupervised deep learning method based on CNN with four convolutional layers with a max-pooling to learn the characteristics of dense and fatty tissues. Unlabeled imaging data were used to carry out unsupervised feature learning based on CNN to segment the mammogram’s breast density regions. In their approach, the input mammogram is divided into sub-images classified into dense or fatty regions. The convolutional layers in the unsupervised parts are trained as auto-encoders, and in the supervised part, the (pretrained) weights and bias terms are fine-tuned using Soft-Max regression 5-fold cross-validation (CV). The accuracy of mammographic texture by sparse convolutional autoencoder (MT-CSAE) and the accuracy of mammographic texture density (MT-density) were 0.57 and 0.59, respectively.

In another study, Dalmış et al. [32] applied a deep learning-based U-net method for segmenting the breast tissue and achieved the average DSC value of 0.897%. Additionally, the study of Lee and Nishikawa [11] proposed a fully convolutional neural network (FCNN) to segment the dense fibroglandular areas on mammographic images automatically. For the evaluation of their method, 455 full-field digital screening mammograms of 58 cases were used. They fine-tuned the ImageNet-pretrained VGG16 [33] for breast density segmentation and estimation. The Percent Density (PD) estimation by their approach showed similarities with BI-RADS density assessment by radiologists with 0.81% for CC view, 0.79% for MLO view, and 0.85% accuracy on average. Automated mammographic breast density estimation using a fully convolutional network [11] that applied FCN techniques carried seven convolution layers compared with our methods is more complicated.

Aly A. Mohamed. [34] presented a modified AlexNets for classifying the BI-RADS II, and BI-RADS III with accuracy (MLO and CC) = 0.92 and classification the (dense) BI-RADS I and BI-RADS II, (non-dense) (BI-RADS III and BI-RADS IV) with AUC (CC and MLO) = 0.95.

In another study, Aly A. Mohamed. [35], a deep learning model using a CNN structure for breast density classification based on the standard categorization of BI-RADS. In addition, two classifiers based a CNN structure and an improved AlexNet model were proposed in [34] to discriminate the breast density categories. Between categories, BI-RADS II and BI-RADS III, by 6-fold CV and 925 images each, with an accuracy of 94% compared to the local institution’s radiological reports. Classification accuracy was reported to increase up to 98% when excluding image data of more inferior quality. Furthermore, the study of Li et al. [36] presented a technique to separate the breast region into two parts, the “dense region” and the “fatty region”. They used deep CNN with three convolution layers, which contained six stages. The first three stages were used as a feature generator, and the second three stages were used as probability predictor. Their dice similarity coefficient is 0.76%, with a 0.94% correlation coefficient. In the study of Li et al. [36], the density is classified into only two classes, whereas in the present study, a classifier divided into four classes is proposed.

In another study, Lehman et al. [22] have demonstrated deep convolutional neural network (DCNN) methods based on ImageNet-pretrained ResNet18 for breast density classification. They applied 41,479 images to classify them into two dense and non-dense classes and achieved 0.87% accuracy.

Dubrovina et al. [37] presented a tissue classification method by supervised CNN framework using a patch-wise approach for CNN training in mammography images. Raw DNN output was recorded as 0.80, and postprocessed DNN output was achieved as 0.81.

Ciritsis et al. [38] applied a DCNN model for breast density classification with 13 convolutional layers, four dense layers in four density classes with two different sets. The first set included 850 MLO, 882 CC, and BI-RADS accuracy was between 71% and 71.7%, dense-vs.-nondense: 88.6–89.9%. The second set included 100 MLO and 100 CC; the accuracy of BIRADS was between 87.4% and 92.2%, dense-vs.-nondense: 96–99%.

Gandomkar et al. [21] investigated Inception-V3 network architecture to process the mammograms and pretrained the system based on ImageNet. Their network achieved an accuracy of 92.0% in high against low-risk classification.

Many breast density classification methods in mammograms have been presented in the literature, but only a small number of studies have achieved accuracy above 90%, which is more complicated than the method presented in this study. In this study, a novel method of representing breast tissue is presented. We are modeling dense tissue distribution to fatty tissue and how this can be utilized to provide the density segmentation classification based on BI-RADS and density percentage. It should be noted that all mammograms were classified according to the BI-RADS density classification system by expert breast radiologists.

The novel and advantageous features of the method proposed in this study are as follows.

- The first adaptation of cGAN in the area of fully automated breast density segmentation in mammograms is developed,
- the breast density percentage classification by the developed multi-class CNN architecture correlated well with BI-RADS density ratings (BI-RADS I, BI-RADS II, BI-RADS III, and BI-RADS IV) using the binary mask segmented in the previous stage (cGAN output) by radiologists,
- a strong correspondence between the output of our automated algorithm and radiologist’s presented breast density measures can be obtained, and
- the proposed approach results in remarkably faster calculation while improving the classification efficiency compare to other methods in the literature

## 3. Methodology

**Figure 2:**
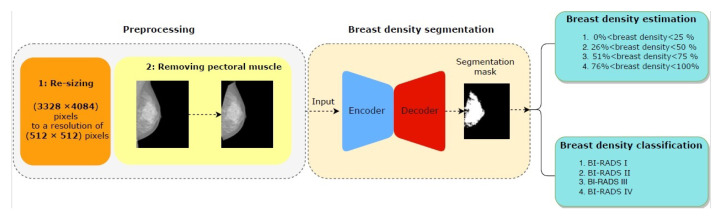
An overview of proposed framework.

This section gives detailed information about the methodology used in this study. The full research methodology is shown in Figure 2, and it is divided into two stages: stage one includes breast mammogram segmentation into background, pectoral muscle, and breast tissue region. The second stage is corresponding to breast density classification based on BI-RADS.

In the first stage, mammograms are prepossessed for removing pectoral muscles. Later, the cGAN input is rescaled by resizing the mammograms to 512×512 pixels, including different breast densities. The processed mammograms are then fed to the proposed cGAN to get a binary mask containing the dense tissue.

In the second step, two methods are used: (1) computing the percentage of breast density by dividing the area’s dense tissues into the breast’s total area, and (2) the output binary mask is down-sampled into 128×128 pixels, which is used to classify breast density in 2 different ways; first, the output of the binary mask is fed to a multi-class CNN to classify the breast density into four classes based on BI-RADS, and second the breast density percentage by traditional method based on thresholding rules is estimated.

Several techniques are applied in this study to prepare the dataset before feeding the mammograms into the proposed model; the stepwise details are explained in the following subsection.

### 3.1. Preprocessing

Some preprocessing operations in the first step are used, such as removing the pectoral muscles and resizing the mammographic images. The high similarity in intensity and the overlap between the pectoral muscle and the glandular tissue can cause false-positive detection of dense tissue area in mammographic images. Therefore, extraction of the pectoral muscle area is applied, which can reduce the false positives [28], as the identification and removal of pectoral muscles play an essential preprocessing step in CAD system [31].

To remove the pectoral muscles, an automatic method described in our preliminary work [39] has been utilized. This method involves three main steps. First, the breast region and the pectoral muscle are segmented from the background, and then secondly, the mammogram orientation is determined. Finally, a region growing segmentation is used for removing the pectoral muscle from the image.

**Figure 3:**
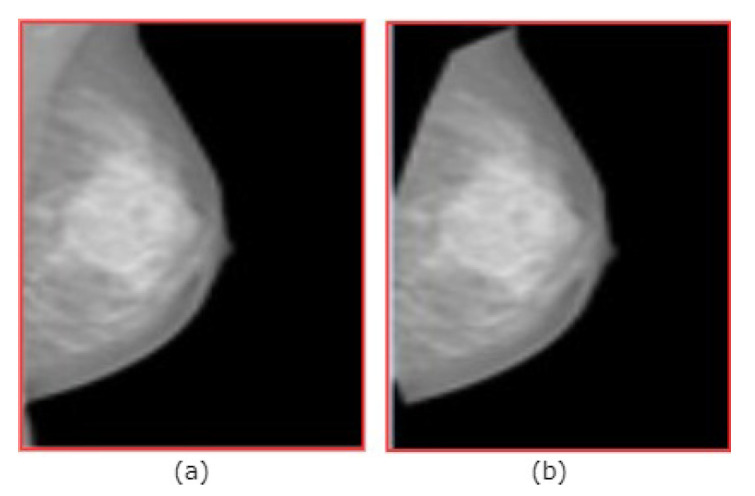
(**a**) Original images. (**b**) After removing pectoral muscle.

The example of removing the pectoral muscles is shown in Figure 3. In addition to reduce the computation time, all mammograms were re-sized from (2560×3328) or (3328×4084) pixels to a resolution of (512×512) pixels (i.e., the resolution yields the best accuracy for the segmentation stage).

### 3.2. Breast Density Segmentation

**Figure 4:**
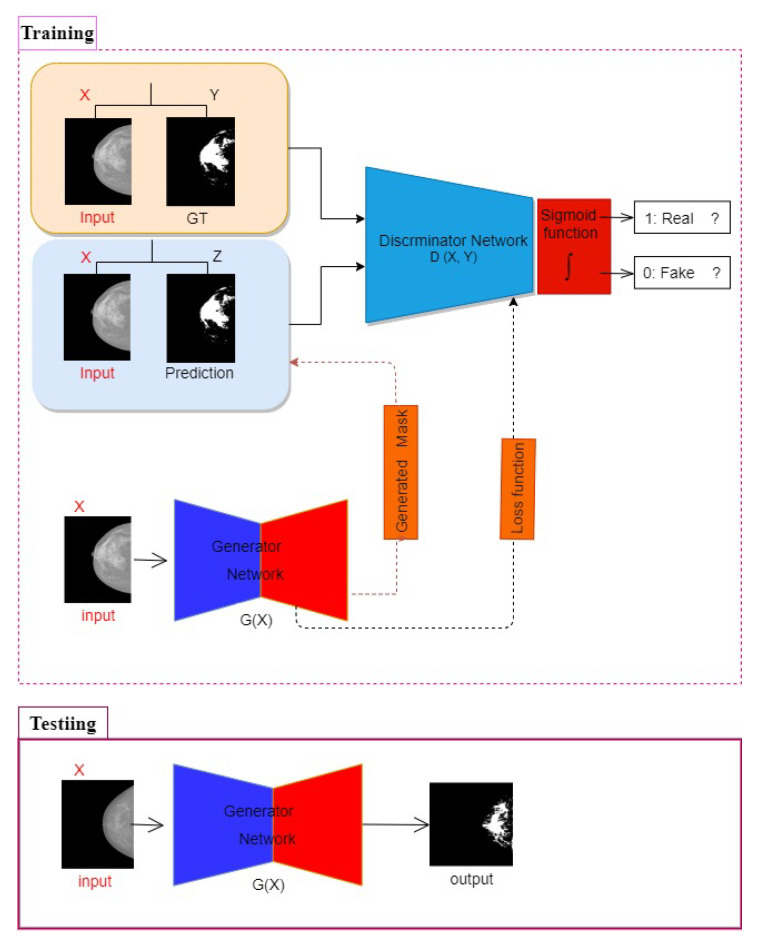
cGAN framework for segmentation.

The proposed framework of breast density segmentation is presented in Figure 4, followed by a summary of each step in the process. For dense tissue segmentation, the conditional cGAN is used, which is proposed by [40]. cGAN is a conditional variation of the GAN, where the generator is instructed to generate a real sample having specific characteristics rather than a generic sample from full distribution [24]. It has been assumed that the cGAN structure is well suited to accurately outline the breast density area, especially when the training data is limited, and our experimental results support our hypothesis. As demonstrated in Figure 4, the cGAN network comprises two main networks: generator and discriminator.

The Generator network G comprises two parts: encoder and decoder layers. The encoder layers help extract the features (e.g., texture, edge, shape, and intensity) from the input images. On the other hand, decoder layers generate a binary mask according to these extracted features.

The Discriminator network D works as a classifier to discern between the generated binary mask and its corresponding ground truth. This adversarial network always tries to enforce the generator network by its working methodology during the training process.

In the model presented in this study, the G network takes a mammographic image and tries to generate a mask image of the areas related to dense tissues (i.e., 0 for non-dense pixels including the background pixels, and 1 for dense tissues pixels). The generator network then generates data latterly fed into a discriminator network. The discriminator D learns a loss function to train this mapping by comparing the ground-truth and the predicted output, but with observing the input image as a condition to improve the network optimization as proposed in [40].

The G network follows an encoder–decoder architecture of U-net with skip connection [41]. The encoder includes downsampling eight convolutional layers. The first layer uses 7×7 convolution to generate 64 feature maps, and the final layer generates 512 feature maps with a 1×1 size. At the same time, the six middle layers are from the pretrained ResNet-101 [41]. In turn, the decoder includes upsampling eight convolutional layers with reverse ordering layers that are similarly structured to the encoder network. A U-net architecture based on skip connections in which each decoder’s input is concatenated to its corresponding convolutional output of the encoder is also used in this study to improve the segmentation performance. On the other hand, the discriminator network consists of 5 convolutional layers. The first layer of the discriminator used 64 filters of 3×3 and a stride of 2×2. The final layer of the discriminator produces 512 feature maps with a size of 62×62, followed by Sigmoid as an activation function. The proposed cGAN model has been trained over a loss function resulting from combining content and adversarial losses. The content loss $L_{c}$ follows a classical approach in which the predicted dense mask is pixel-wise compared to the corresponding one from ground-truth. For this loss, three loss functions are tested: Mean Square Error (MSE), Dice, and structural similarity index (SSIM). The adversarial loss depends on the real/fake prediction of the discriminator over the ground-truth and the predicted foreground mask with observing the input image. Assume the input mammography image is *x*, *y* the ground truth mask, *z* a random variable, λ an empirical weighting factor, G(x,z) and D(x,G(x,z)) the outputs of *G* and *D*, respectively. Thus, the training process of this cGAN can be expressed as an optimization of the following objective function presented in Equation (1), which mathematically describes the training of cGAN.

(1) $$G^{*}=arg\min_{G}\max_{D}L_{cGAN}\left(D,G\right)+\lambda L_{c}\left(G\right)$$

where λ=10, and $\;L_{cGAN}\left(D,G\right)$, the binary cross entropy (BCE) of the adversarial, can be computed as shown in Equation (2):

(2) $$L_{cGAN}\left(\;D,G\right)=E_{x,y}(\log\left(D\left(x,y\right)\right)+\;E_{x,z}(\log(1-D(x,z))$$

In Equation (2), the first term is the entropy of the discriminator *D* with real data (i.e., the input image is *x*, and the ground-truth is *y*, both images are concatenated). The second term is entropy with a fake input data (i.e., the input image is *x* and the generated image is *z*, both images are concatenated) passes through the generator, which is then passed through the discriminator to identify the fakeness (i.e., the log probability that the data from generated is fake if it equals to 0), and the content loss function computed between *z* and its corresponding ground-truth *y*, can be defined using Equation (3):

(3) $$L_{c}\left(G\right)=f\left(y,G\left(x,z\right)\right),$$

where *f* is MSE, Dice, or SSIM loss functions. The MSE loss function can be computed using Equation (4):

(4) $$L_{MSE}\left(y,G\left(x,z\right)\right)=\frac{1}{N}\;\sum_{j=1}^{N}{∥y-G\left(x,z\right)∥}_{2}$$

where *N* is the number of the pixels per input image and $L_{Dice}\left(y,G\left(x,z\right)\right)$ is the dice loss of the predicted mask concerning ground truth, which is defined using Equation (5):

(5) $$L_{Dice}\left(y,z\right)=1-\frac{2|y\times G(x,z)|}{\left|y|+|G(x,z)\right|}$$

The SSIM [42] considers contrast, luminance, and structure to determine the similarity between two images. SSIM can be calculated using Equations (6) and (7).

(6) $$SSIM\left(y,z\right)=1-\frac{\left(2\mu_{y}\mu_{z}\right)+c_{1})\left(2\sigma_{yz}+c_{2}\right)}{\left(\mu_{y}^{2}+\mu_{z}^{2}+c_{1}\right)(\sigma_{y}^{2}+\sigma_{z}^{2}+c_{2)}}$$

(7) $$\sigma_{yz}=\frac{1}{T-1}\sum_{i=0}^{T}\left(y_{i}-\mu_{y}\right)\left(z_{i}-\mu_{z}\right)$$

where *y* and *z* are the ground-truth and generated images, respectively; $\;\mu_{y}$ is the average of *y*; and $\;\mu_{z}$ is the average of *z* (are local measures of the mean of the ground-truth and generated images). $\;\sigma_{y}$ is the standard deviation of *y*, $\;\sigma_{z}$ is the standard deviation of *z*. $\;\sigma_{yz}$ is the local measures of the correlation between two images, $\;\sigma_{y}^{2}$ and $\;\sigma_{z}^{2}$ are the local measures of the variance of the two images; and $\;C_{1}={\left(k_{1}L\right)}^{2}$, $\;C_{2}={\left(k_{2}L\right)}^{2}$ are some predefined constants that are two variables to stabilise the division with small denominators, *L* is the dynamic range of the pixel values (typically this is 255), $k_{1}$ = 0.01 and $k_{2}$ = 0.03 by default. *T* is the total number of pixels in each image. The optimization process of *G* tries to minimize both expected values, i.e., the *D* values should approach 1.0 (correct tumor segmentation), and the content loss $L_{c}$ should approach 0.0 (generated masks equal to ground truth). Both terms of generator loss enforce the proper optimization of *G*: the dice loss term fosters a rough prediction of the mask shape (central tumor area), while the adversarial term fosters an accurate prediction of the mask outline (tumor borders). Neglecting either of the two terms may lead to very poor segmentation results or slow learning speed.

During the training process, the discriminator tries to maximize the function presented in Equation (1), while the task of the generator is precisely the opposite that tries to minimize the function presented in Equation (1).

For our experiments, an Adam optimizer [29] with a learning rate of 0.0002 and batch size equal to 4, in addition to an optimal number of epochs equals 200, has been used.

Two main training and testing procedures can be distinguished. During training in a supervised mode, the classifier learns to distinguish between fatty and dense pixels from manually annotated images, whereas in testing, the classifier assigns a fatty or dense tissue label to each pixel of the input image [6].

**Figure 5:**
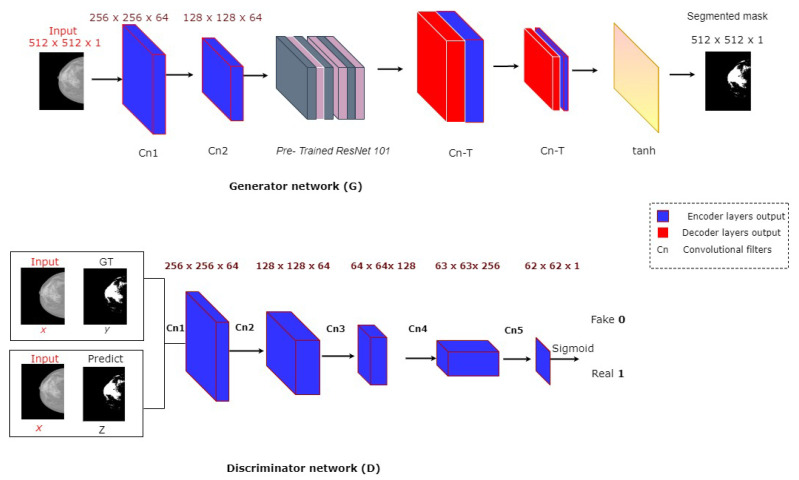
cGAN framework for breast density segmentation.

Figure 4 shows the framework of the proposed method for breast density segmentation. Moreover, a pretrained ResNet-101 was used as a base feature extractor, which is illustrated in Figure 5.

### 3.3. Breast Density Classification

For breast density classification, two techniques, including a traditional method and a CNN-based method, have been used; each method has four output classes described in the following sections.

#### 3.3.1. Breast Density Percentage Estimation Based on Traditional Method

Percent density obtained from mammographic images refers to the ratio of the area of dense tissue present in a mammogram to the total area of the breast. For the traditional method, we perform five stages:

- First, we resize the generated mask images to the same resolution of the input mammography.
- We express the breast region area by the number of non-zero pixels in the mammogram images.
- We then count the non-zero pixels in the generated mask for expressing about the area dense tissues.
- Computing the ratio between the area of dense tissues and the area of the breast region to estimate the breast density in the input image.
- Finally by the thresholding rules defined in the BI-RADS density standard, we classify the breast density to 4 categories: (0% < BDE <25%), (26% < BDE < 50%), (51% < BDE < 75%), (76% < BDE < 100%).

#### 3.3.2. Breast Density Classification Based on a CNN

Figure 5 shows the CNN architecture for breast density classification (four classes correspond to the BI-RADS categories: fatty, scattered fibroglandular density, heterogeneously dense, and extremely dense) using the binary masks obtained from the cGAN.

Most methods which are attempted to categorize the breast density have computational complexity.

**Figure 6:**
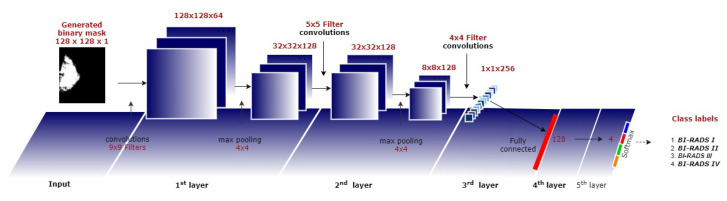
Proposed CNN architecture used for breast density classification (second technique).

As shown in Figure 6, the proposed CNN technique consists of three convolutions layers with kernel sizes 9×9, 5×5, and 4×4, respectively, and two fully connected (FC) layers. The first two convolutions layers are followed by 4×4 max-pooling with stride 4×4. The last convolution layer’s output is flattened and then fed into the first FC layer with 128 neurons. These four layers use ReLU as an activation function. A dropout of 0.5 is used to reduce overfitting in the first FC layer. Finally, the last FC layer with four neurons applies the soft-max function to generate the input binary mask’s final membership degree to each class. A weighted categorical cross-entropy loss is used to avoid the problem of an unbalanced dataset. The class weight is one minus the ratio of samples per class to the total number of samples.

The RMSProp is applied for optimizing the model with a learning rate of 0.001, a momentum of 0.9, and a batch size of 16. The network is trained from scratch, and the weights of 5 layers are randomly initialized. The best architecture, number of layers, filters per layer, and neurons in FC layers during training were found experimentally.

## 4. Experimental Results

### 4.1. Dataset

**INbreast dataset (http://medicalresearch.inescporto.pt/breastcancer/index.php/Get_INbreast_Database/)**:

The initial results presented in this section are based on the INbreast dataset [43]. It is a publicly available database that is 2-dimensional (2D) and includes MLO and CC mammographic images of 115 patients (410 mammograms). Every patient has 4 mammographic images, which consists of MLO-right, MLO-left, CC-right, and CC-left.

It has a ground truth for mass location, mass type, and breast density classification label. Breast density classification in INbreast was prepared based on breast imaging reporting and data method (BI-RADS) standard (BI-RADS I, BI-RADS II, BI-RADS III, and BI-RADS IV). The image size of mammogram is 3328×4084 or 2560×3328 pixels. Note that the INbreast dataset does not have the ground truth binary masks for the breast density segmentation. Thus, we have annotated the images with the cooperation of radiologists experts in breast cancer. The INbreast dataset has 115 patients divided into 82 patients (80% of the total images) as a training set and 33 patients (20% of the total images) as a test set. To train the proposed CNN network, we applied the “Holdout cross-validation method” on the images of the 82 patients to divide it into 80% for training and the rest for validation. The “Holdout cross-validation method” ensures that the images are randomly divided into training and validation sets without any intersection between them to guarantee a fair evaluation.

In the holdout cross-validation, (33%) of data belong to BI-RADS I with 108 images of 27 patients, (35%) of data belong to BI-RADS V with 116 images of 29 patients, (25%) of data belong to BI-RADS III with 80 images of 20 patients, and only (7%) of data belong to BI-RADS IV with 22 images of 6 patients. Table 1 shows the INbreast dataset distribution for training and test sets.

One of this study’s principal goals is to create a robust model that generalizes well to new data and uses images of patients not initially included in the training stage. For testing, 33 patients have been used as a control/test set for evaluating the performance of the trained deep models for dense tissue segmentation and breast density classification. Thus, our control/test set can serve as a proxy for new data.

Table 1 breaks down breast density variety from the INbreast dataset. The distribution of breast densities variety across the four classes is shown in Table 1. As shown, it can be seen that the dataset is highly imbalanced with the lowest percentage (7%) of data belong to BI-RADS IV, and the highest percentage of data (35%) belong to BI-RADS II.

Indeed, the INbreast dataset used for training the segmentation model does not have the ground truth (i.e., binary masks) for the dense tissue segmentation. However, it has an annotation for the images with the corresponding class of BI-RADS. The lack of ground truth for breast density assessment is a limitation. Therefore, the dense regions in the INbreast dataset images are segmented by two radiologists from Hospital Sant-Joan de Reus (Reus Sant-Joan Hospital located in Tarragona province, Spain). Pixel-wise *logical-AND* was applied on the binary masks generated by the two radiologists to generate the ground truth, meaning the two radiologists have to agree about the dense tissues in the same mammogram. Assume we have a binary mask for each mammogram generated by each radiologist. If the same pixel has a value 1 in both binary masks, it will be 1 in the final ground-truth image.

### 4.2. Implementation Details

The proposed method was applied using Python v.3.5 with PyTorch library (https://pytorch.org), running on a 64-bit Ubuntu operating system, a 3.4 GHz Intel Core-i7 CPU with 16 GB of RAM, and NIVIDA GTX 1070 GPU with 8 GB of video RAM.

#### Evaluation Metrics of Breast Density

The terms and formulas involved in evaluating the results of breast density classification are described in (8) to (11):

(8) $$\mathrm{Accuracy}=\frac{Correctpredictions}{Totalpredictions}=\frac{\left(TP+TN\right)}{\left(TP+TN+FP+FN\right)}$$

(9) $$\mathrm{Precision}=\frac{Truepositive}{Predictedpositive}=\frac{TP}{\left(TP+FP\right)}$$

(10) $$\mathrm{Sensitivity}=True-PositiveRate=Recall=\frac{Truepositive}{Actualpositive}=\frac{TP}{TP+FN}$$

(11) $$\mathrm{Specificity}=True-NegativeRate=\frac{TN}{TN+FP}$$

In this work, we need to compute *TP*, *TN*, *FP*, and *TN* for a multi-class problem that has one score for each class and counts any other class as a negative. For example, in our case, we have four classes (1, 2, 3, 4); thus, *TP*, *FN*, *FP*, and *TN* for C1 can be calculated as

- True positive (*TP*) instances are gold standard class 1 predicted as class 1
- False-negative (*FN*) instances are gold standard class 1 predicted as class 2, 3, or 4
- False-positive (*FP*) instances are gold standard class 2, 3, or 4 predicted as class 1
- True negative (*TN*) instances are gold standard class 2,3 or 4 predicted as class 2, 3, or 4 (here errors do not matter as long as class 1 is not involved)

Similarly, for the three other classes C2, C3, and C4, we can compute TP, FN, FP, and TN.

### 4.3. Breast Density Segmentation

It is of high importance to accurately estimate the breast density for achieving proper dense tissue segmentation. In the first experiment, three variations of the cGAN-UNet network with the different content loss function $L_{c}$ are evaluated: MSE, Dice, and SSIM.

For the quantitative analysis, the quality of the dense region’s segmentation is measured using three evaluation metrics: accuracy, Dice coefficient or F1 score (DSC), and Jaccard index (JI). Quantitative results are shown in Table 2. Note that we separately computed the metric value of the images of class 1 (C1), class 2 (C2), class 3 (C3), and class 4 (C4), while “all” refers to the metric value of all images of the testing set. Table 2 contains the summary of all the methods tested over the three evaluation metrics; Accuracy, Jaccard index, and Dice over the segmented images.

As shown in Table 2, the cGAN-UNet with Dice provides the best accurate dense regions segmentation, among the other proposed models with an accuracy of 98%. cGAN-UNet with Dice yields a significant improvement of 7% and 11% with DSC and JI, respectively, compared to the cGAN-UNet with MSE, which reflects the highest similarity between the ground truth and the predicted segmentation. The segmentation performance of cGAN networks for each class of the INbreast dataset, using Dice, gives the best results for the four classes in accuracy. In turn, cGAN-UNet with an SSIM loss is the second-best model yielding an accuracy of 96%, DSC of 79%, and JI of 65%). The lowest overall dense tissue segmentation performance with an accuracy of 80% has been obtained by cGAN-UNet using the MSE content loss function, in which the values of DSC and Jaccard scores achieved are 80% and 67%, respectively. The results with the three variations indicate that DSC and SSIM as loss functions help the adversarial network in training the generative network better than MSE. The poor performance of the MSE loss function is because it is prone to outliers. After all, it uses the Mean in computing each error value. In turn, the DSC and SSIM loss increases the similarity between the dense segmented regions and the ground truth.

**Figure 7:**
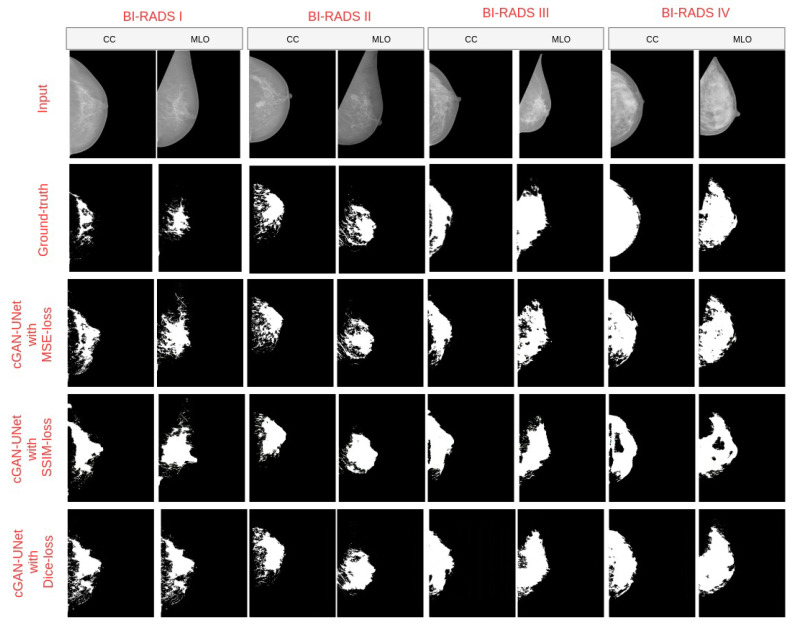
Breast density segmentation result of three models with the INbreast dataset. (Row 1) original images. (Row 2) Ground truth. (Row 3) Result of cGAN-UNet with dice-loss. (Row 4) Result of cGAN-UNet with myssim loss. (Row 5) Result of cGAN-UNet. (Col 1) BI-RADS I from two different views (CC and MLO). (Col 2) BI-RADS II. (Col 3) BI-RADS III. (Col 4) BI-RADS IV.

Figure 7 supports the quantitative results of Table 2, as the segmented images resulted in the cGAN-UNet with an Dice loss accurately segmented the dense regions, including the small regions and preserving the small details and boundaries of the dense tissues. To assess the proposed model (i.e., cGAN-UNet with a Dice loss providing the best results in Table 2) and to show its effectiveness, it was compared against state-of-the-art segmentation models that are commonly used for semantic segmentation based on deep learning models such as FCN8 [44], FCN32 [44], and Vgg-Segnet [45].

The results of this assessment are shown in Table 3. All models are trained and tested on the INbreast dataset. It is noteworthy that these results show that the proposed model developed in this study outperformed the other models in terms of Sensitivity, Specificity, Precision, and DSC score. The FCN-32 achieved the worst results among the evaluated methods with a DSC score of 58%. This network consists of 32 convolutional layers that need many images to be appropriately trained; however, most medical datasets lack enough images as the main difficulty. Therefore, the FCN-8 model with eight layers achieved an improvement of 14% in the DSC score compared to FCN-32. Besides, the Vgg-Segnet network provided acceptable results with a DSC of 73%. In turn, cGAN-UNet with the Dice loss yields an improvement of 15% in the DSC score compared to the Vgg-Segnet. Furthermore, it yields an improvement of 12% of Precision better than the best second method, FCN-8. Regarding Sensitivity, cGAN-UNet achieved the best result among the four deep models with an improvement of12% compared to Vgg-Segnet, the second-best model. For Specificity, the four methods provide very high Specificity; however, our model based on cGAN yields the lowest Specificity of 98.5% among them only 1.2% lower than FCN-8 and FCN-32 models.

In conclusion, cGAN-UNet, with its variations proposed in this study, can learn the statistical invariant features (texture, color, etc.) of an input image and then generate nearly segmented images, which look like the ground-truth image. However, this study’s segmentation model contains about 13,607,043 parameters for tuning the generator part in the cGAN network. The method developed in this study is fast in both training, i.e., around 30 s per epoch (326 images) and predicting, around seven images per second. That is surprisingly 10–15 times faster than the FCN-32 mode and 7–8 times faster than the FCN-8 model.

### 4.4. Breast Density Classification

This paper proposed two different classification techniques with four output classes. In the first technique, breast density is classified into four categories by traditional method and breast density percentage estimated by thresholding rules. Whereas in the second method, a CNN model is applied on the generated binary masks.

A confusion matrix of the traditional method is provided in Table 4. For the traditional method, the overall accuracy of breast density percentage classification based on thresholding rules is 80%. For class-1 (BI-RADS I), the traditional method properly classified 77% of the images. The traditional method of breast density gives the lowest accuracy with class-2. This happens as there is a high correlation between class-1 and class-2. For class-3 (BI-RADS III) and class-4 (BI-RADS IV), we achieved classification rates of 90% and 84%, respectively.

For the proposed breast density classification based on a trained CNN network, two experiments have been applied: one for the classification of the imbalance dataset (The training data without augmentation), and the other one for a balanced dataset (the training data after augmentation). The network have been tested with two different image sizes, 64×64 and 128×128. Note that the input to the CNN-based method is the binary image generated by the segmentation model. As explained in the above subsection of evaluating breast density segmentation, the cGAN-UNet with a dice loss yields the best segmentation results. The results of this network were used to classify the breast density in mammography.

Due to the imbalanced data set, deep learning classification performance may decrease. Thus, to overcome the imbalanced number of training images, we have done data augmentation by applying “Illumination change”, “scaling”, and “flipping”, which yields 798 images for each class, is shown in Table 5. We applied the augmentation techniques on the training set only with 326 images of 82 patients. In turn, the same test set of 84 images of 33 patients is used for evaluating the two trained CNN networks.

The evaluation of CNN-classification in terms of accuracy, precision, sensitivity, and specificity have been applied on imbalanced and balanced datasets with two different sizes of input images (64 × 64 and 128 × 128) are detailed in Table 6.

As shown in Table 6, the CNN-based classification method’s performance is higher when using a balanced dataset than the imbalanced data set in terms of the four evaluation measures. The lowest overall accuracy of CNN classifiers for an imbalanced dataset with an input image size of 128 is 90.29%. In turn, the classification rate after applying augmentation to different sizes of input images of (128 × 128 and 64×64) are 98.75% and 98.62%, respectively. However, the CNN-based classification method’s overall accuracy is with a balanced dataset and a 128×128 image size is 98.75%, with an improvement of 0.13% higher than the classification result with a 64×64 image size.

The confusion matrix of the CNN-based classification method with different input images is shown in Table 7. As shown, by using the balanced dataset (3192 images) with an image size of 128×128, the CNN classifier can correctly predict the class IV with 100% accuracy as class-4 contains the high dense masks. In contrast, when the image size is changed to 64×64, the CNN classifier can adequately predict class I and class IV with an accuracy of 100% and class III with an accuracy of 98%. The CNN-based classification method results show how data augmentation and constructing balanced datasets can improve the overall classification accuracy. Besides, some important objectives, such as minimizing the complexity (for example, in the LIBRA handcrafted method, they combined 86 features; in turn, in the FCN technique [11], seven convolution layers were used, which is more complicated than our network structure with only four layers), maximizing classification accuracy, maximizing true-positive rate, and minimizing false-positive rates are achieved in these classification approaches.

For a quantitative correlation, in terms of accuracy, the performance of our proposed algorithm was compared against state-of-the-art breast density classification. A summary of representative studies can be found in Table 8. The accuracy, classification method, number of datasets, and number of density categories are represented in Table 8. As shown, the performance of the proposed technique outperformed the state of arts with an overall accuracy of 98.75%.

## 5. Conclusions

Breast density can undoubtedly affect the accuracy of routine breast cancer detection methods, such as screening mammography. Therefore, it would be a breast cancer diagnostic dilemma for women with dense breast tissue (approximately 50 percent of women) [46]. This study aims to develop an innovative and accurate method to segment and classify the breast density based on BI-rads standard. Traditional breast density segmentation and classification methods are cumbersome tasks and have a high possibility of false positives. The efficacy of a fully automated algorithm for breast density segmentation and classification in digital mammography is proposed and substantiated by presenting three versions of cGAN networks for segmentation and two different classification methods. In our experiments, mammograms of 115 patients (410 images) from the INbreast dataset were used. With the breast density segmentation task, our method achieved an accuracy, Dice coefficient, Jaccard index of 98%, 88%, and 78%, respectively. With the density classification task, our method obtained precision, sensitivity, and specificity of 97.85%, 97.85%, and 99.28%, respectively. A strong correlation can be obtained between the computerized algorithm’s output and the radiologist’s estimated breast density. This observation justifies that the proposed methods in this study have a strong positive relationship with the radiologist manual classification and is competitive with reported correlation coefficients from the literature, e.g., 0.63 [31], 0.70 [35], 0.85 [36], and 0.85 [11]. The most notable limitation of this study is that only one dataset is used. For future developments, more datasets need to be utilized; however, this dataset’s ground-truth is prepared by doctors, experts, and radiologists of the Hospital Universitari Sant-Joan de Reus, Spain, via developing our GUI in MATLAB to help the radiologists to annotate the images. It is believed that artificial intelligence is capable of surpassing human experts in breast density prediction. The future work of this research is to transpose our fully automated PD% estimation techniques into the robust computer-aided breast density analyzer appraisal tool for use in clinical practice. 

## Figures and Tables

**Table 1:** Distribution of densities across imbalanced INbreast dataset (before augmentation).

	I	II	III	IV
**Total dataset** **(410 images)**	33%, 136 images	35%, 146 images	25%, 100 images	7%, 28 images
	from 38 patients	from 41 patients	from 25 patients	from 8 patients
**Training and validation subset** **(328 images)**	33%, 108 images	35%, 116 images	25%, 80 images	7%, 22 images
	from 27 patients	from 29 patients	from 20 patients	from 6 patients
**Test subset**	33%, 27 images	35%, 29 images	25%, 20 images	7%, 6 images
**(82 images)**	from 11 patients	from 12 patients	from 5 patients	from 2 patients

**Table 2:** Accuracy, DSC, and J I with the cGAN-UNet, cGAN-UNet-SSIM-loss, and cGAN-UNet-dice-loss evaluated on the testing set of the INBreast dataset for breast density segmentation (C1 = Class1, C2 = Class2, C3 = Class3, C4 = Class4).

Model	Accuracy					DSC					J I				
	C1	C2	C3	C4	All	C1	C2	C3	C4	All	C1	C2	C3	C4	All
**cGAN-Unet with Dice-loss**	0.98	0.99	0.99	0.99	**0.98**	0.66	0.90	0.95	0.95	**0.88**	0.50	0.82	0.91	0.91	**0.78**
cGAN-Unet with SSIM-loss	0.94	0.98	0.98	0.96	0.96	0.54	0.86	0.91	0.85	0.79	0.37	0.75	0.83	0.74	0.65
cGAN-Unet with MSE-loss	0.68	0.84	0.93	0.95	0.80	0.53	0.85	0.94	0.97	0.81	0.36	0.74	0.90	0.94	0.67

**Table 3:** Summary of Sensitivity, Specificity, FPR, FNR, Precision, and DSC with the cGAN-UNet-MSE-loss, FCN8, FCN32, and VGG-SegNet methods evaluated on the testing set of the INBreast dataset for breast density segmentation.

Methods	Sensitivity	Specificity	Precision	DSC
**cGAN-UNet with Dice-loss**	**0.957**	**0.985**	**0.81**	**0.88**
FCN-8 [44]	0.748	0.997	0.69	0.72
FCN-32 [44]	0.5724	0.997	0.59	0.58
Vgg-Segnet [45]	0.832	0.996	0.66	0.73

**Table 4:** Confusion matrix of breast density estimated based on thresholding rules.

	Class	Predicted Label			
Ground truth		I	II	III	IV
	I	**0.77**	0.20	0.03	0
	II	0	**0.76**	0.24	0
	III	0	0	**0.90**	0.10
	IV	0	0	0.16	**0.84**

**Table 5:** Distribution of densities across balanced INbreast dataset (after augmentation).

	I	II	III	IV
**Total dataset** **(3192 images)**	25% (798 images)	25% (798 images)	25% (798 images)	25% (798 images)
**Training subset** **(2552 images)**	25% (638 images)	25% (638 images )	25% (638 images)	25% (638 images)
**Validation subset (640 images)**	25% (160 images)	25% (160 images)	25% (160 images)	25% (160 images)
**Test subset**	27 images	29 images	20 images	6 images
**(82 images)**	of 11 patients	of 12 patients	of 5 patients	of 2 patients

**Table 6:** Accuracy, Precision, Sensitivity and Specificity of CNN-based classification method on imbalanced and balance dataset with two different sizes of input images: 64×64 and 128×128.

	Size of Input Images	Accuracy	Precision	Sensitivity	Specificity
Imbalanced dataset (410 images)	128 × 128	90.29	90.29	90.29	96.76
	64 × 64	94.95	94.17	94.17	98.05
Balanced dataset (3192 images)	128 × 128	**98.75**	97.5	97.5	99.16
	64 × 64	98.62	**97.85**	**97.85**	**99.28**

**Table 7:** Confusion matrix of breast density by CNN-based classification method.

		Size of Input Images	Class	Predicted Label by CNN			
Ground truth	Imbalanced dataset (410 images)	128×128		I	II	III	IV
			I	1.0	0.0	0.0	0.0
			II	0.03	0.93	0.03	0.0
			III	0.0	0.12	0.77	0.12
			IV	0.0	0.0	0.33	0.67
		64×64	I	0.98	0.0	0.0	0.0
			II	0.0	0.96	0.04	0.0
			III	0.0	0.0	1.0	0.0
			IV	0.0	0.0	0.57	0.43
	Balanced dataset (3192 images)	128×128	I	1.0	0.0	0.0	0.0
			II	0.0	0.97	0.03	0.0
			III	0.05	0.0	0.95	0.0
			IV	0.0	0.0	0.0	1.0
		64×64	I	1.0	0.0	0.0	0.0
			II	0.07	0.93	0.0	0.0
			III	0.0	0.0	0.97	0.03
			IV	0.0	0.0	0.0	1.0

**Table 8:** Accuracy of different breast density classification in terms of their best results.

Study	Year	Method	No. of Images	No. of Density Category	Accuracy (%)
Volpara software [27]	2010	Hand-crafted	2217	Dense and Fatty	94.0
LIBRA software [4]	2012	Hand-crafted	324	4 Classes	81.0
Lehman et al. [22]	2018	Deep Learning	41479	Dense and Non-dense	87.0
Lee and Nishikawa [11]	2018	Deep Learning	455	4 Classes	85.0
Mohamed et al. [35]	2018	Deep Learning	925	BI-RADS II and BI-RADS III	94.0
Dubrovina et al. [37]	2018	Deep Learning	40	4 Classes	80.0
Gandomkar et al. [21]	2019	Deep Learning	3813	Dense and Fatty	92.0
**Our proposed CNN**	**2020**	**Deep Learning**	**410**	**4 Classes**	**98.75**
